# Personalized bundle recommendation using preference elicitation and the Choquet integral

**DOI:** 10.3389/frai.2024.1346684

**Published:** 2024-02-14

**Authors:** Erich Robbi, Marco Bronzini, Paolo Viappiani, Andrea Passerini

**Affiliations:** ^1^Department of Information Engineering and Computer Science, University of Trento, Trento, Italy; ^2^Ipazia S.p.A., Milan, Italy; ^3^CNRS, Université Paris-Dauphine, PSL, LAMSADE, Paris, France

**Keywords:** bundle recommendation, Choquet integral, preference elicitation, environmental footprint, explainable recommender systems

## Abstract

Bundle recommendation aims to generate bundles of associated products that users tend to consume as a whole under certain circumstances. Modeling the bundle utility for users is a non-trivial task, as it requires to account for the potential interdependencies between bundle attributes. To address this challenge, we introduce a new preference-based approach for bundle recommendation exploiting the Choquet integral. This allows us to formalize preferences for coalitions of environmental-related attributes, thus recommending product bundles accounting for synergies among product attributes. An experimental evaluation of a dataset of local food products in Northern Italy shows how the Choquet integral allows the natural formalization of a sensible notion of environmental friendliness and that standard approaches based on weighted sums of attributes end up recommending bundles with lower environmental friendliness even if weights are explicitly learned to maximize it. We further show how preference elicitation strategies can be leveraged to acquire weights of the Choquet integral from user feedback in terms of preferences over candidate bundles, and show how a handful of queries allow to recommend optimal bundles for a diverse set of user prototypes.

## 1 Introduction

The task of bundle recommendation consists of generating sets of related products that users tend to consume together in specific situations. A recommendation system based on bundles offers various advantages, ranging from improving user experience through non-trivial complementary items to boosting sales revenue for sellers via cross-selling (Sun et al., 2022) or operational efficiency. To address this task, researchers have proposed a wide variety of approaches, ranging from collaborative filtering (Beladev et al., 2016) and data mining (Fang et al., 2018) to factorization (Cao et al., 2017) and graph neural networks (Deng et al., 2020). However, these solutions rely on historical data to acquire user utilities and cannot be applied to model the preferences of novel users. To the best of our knowledge, the only work addressing preference elicitation for bundles of products (Dragone et al., 2018) models user preferences as linear combinations of bundle properties. This strategy is sub-optimal in that it neglects dependencies between properties in characterizing bundle quality.

Environmental friendliness is a prototypical example of a quality measure that cannot be modeled in terms of a linear combination of properties. Human activities are causing irreversible environmental effects, such as climate change and loss of biodiversity (Rockström et al., 2009). The consumption of products also contributes significantly to individuals' ecological footprint. The production and consumption of more environmentally friendly products is an essential step toward achieving more sustainable lifestyles. Nowadays, the importance of these concepts is being recognized in all areas of human activity, spanning from manufacturing to transportation. Despite this growing awareness, sustainability remains relatively new in the IT sector and is even less emphasized in the field of recommendation systems. The few studies addressing the sustainability of recommendations (Tomkins et al., 2018; Pachot et al., 2021; Merinov et al., 2022) focus on single-item recommendations.

In this study, we introduce a sensible notion of environmental friendliness that depends on bundle attributes in a non-linear way, and show how this notion can be easily formalized in terms of the Choquet integral (Grabisch and Labreuche, 2008), a generalization of the weighted sum that enables the definition of preferences for coalitions of attributes. The Choquet integral is a non-linear aggregation function that is attractive for preference modeling because it can model different kinds of interactions between criteria, and includes many aggregators as special cases (e.g., linear additive models, min, max, and any other order statistics, leximin and leximax, and OWA and WOWA). The Choquet integral has received a lot of attention in the last two decades in the field of decision theory (Grabisch and Labreuche, 2008) and is now widely used in practical decision-making. The interest in using Choquet has driven the development of incremental methods for eliciting the parameters of a preference model based on the Choquet integral (Benabbou et al., 2017) or machine learning methods for learning these parameters from data (Tehrani et al., 2012). However, to the best of our knowledge, it has never been used to model preferences for bundles of products, nor to model the environmental friendliness of a recommendation.

We demonstrate that the Choquet integral can be used to formalize the notion of environmental friendliness of product bundles by modeling the synergies of environmental-related bundle attributes, such as being located in the same warehouse and having the same conservation method. Our experiments show that, when used to recommend bundles of food products, our approach consistently recommends bundles with a higher environmental friendliness score than those that could be selected using more conventional linear methods such as the weighted sum. This is the case even when the weights of the weighted sum are learned to maximize the environmental friendliness score on a training set of candidate bundles. This underscores the significance of the Choquet integral in fully encapsulating the non-linear characteristics of this concept.

Additionally, we show how to leverage a recently proposed preference elicitation strategy (Benabbou et al., 2017) to learn the capacities of the Choquet integral from pairwise queries specified as pairs of candidate bundles. Such strategy employs the minimax regret as the decision criterion, which provides recommendations that reduce the regret to the greatest extent possible under parameter uncertainty, so that the worst-case scenario is minimized (i.e., when suggesting an alternative *x* rather than one of the adversary's options).

An experimental evaluation over simulated users representing prototypical preference patterns (such as a healthy or a net-zero persona) shows how a handful of queries is sufficient to correctly acquire user preferences and recommend optimal personalized bundles. A crucial property of the Choquet integral is that, when formalized in terms of Möbius masses, it provides a natural explanation for the bundle utility in terms of the significance of coalitions of attributes, regardless of the importance of its sub-coalitions.

## 2 Materials and methods

In this section, we first introduce the background concepts for our study: bundle recommendation, the Choquet integral, and preference elicitation (Section 2.1). In Section 2.2, we demonstrate the advantages of adopting the Choquet integral in bundle recommendation through a motivating example. Afterwards, Section 2.3 describe our approach of using the Choquet integral for recommending product bundles.

### 2.1 Background

#### 2.1.1 Bundle recommendation

Bundle recommendation can be defined as the problem of selecting the best group of items from a potentially very large dataset according to some user preferences. This type of recommendation method can involve a series of tasks such as detecting, completing, and ranking bundles, as well as generating bundle explanations and bundle auto-naming (Sun et al., 2022). Bundle recommendation systems can exploit different approaches (Sun et al., 2022):

1. *Constraint-based methods*: Early studies minimize the cost (Garfinkel et al., 2006) or maximize the expected reward revenue of a bundle in e-commerce (Zhu et al., 2014; Beladev et al., 2016). Other methods (Xie et al., 2010; Zanker et al., 2010; Liu et al., 2017) combine constraints (e.g., price, ratings, and user preference) for travel package recommendations.
2. *Data mining-based methods*. Association rule mining is utilized by Fang et al. (2018); Guo-rong and Xi-zheng (2006) for bundle generation and recommendation. In Beheshtian-Ardakani et al. (2018), *K*-means, Apriori algorithm, and SVM are adopted to form and recommend bundles.
3. *Preference elicitation-based methods*. This framework is proposed (Xie et al., 2014; Dragone et al., 2018) to learn utility functions for capturing user preference among various features (e.g., cost and quality) over bundles via user feedback.
4. *Factorization-based methods*. Factorization can be used to jointly factorize user-item, user-bundle interaction matrices and item-item-bundle co-occurrence matrices, to capture user preference over items and bundles (Cao et al., 2017).
5. *Sequence-based neural methods*. The study by Kouki et al. (2019) proposed to combine product hierarchy with transaction data or domain knowledge to identify bundle candidates which are then ranked via an LSTM (Sun et al., 2019)-based deep similarity model.
6. *Attention-based methods*. A factorized attention network can be exploited to aggregate items in a bundle to represent the bundle and jointly model user–bundle and user–item interactions (Chen et al., 2019).
7. *Graph-based neural methods*. Graph convolutional network can be used on the user–item–bundle tripartite graph and perform both item and bundle recommendation tasks for a mutual enhancement (Deng et al., 2020). Graph-based representation can also be used to generate a personalized trip, recommending a list of points of interest maximizing the user travel experience (Chen et al., 2023a).
8. *Reinforcement learning methods*. Chen et al. (2023b) proposed a trip recommendation system based on multi-objective reinforcement learning, formalizing the recommendation problem as a Markov Decision Process (MDP) enhanced with sequential, geographic, and order information to learn the user's context.

Our approach can be seen as a preference elicitation method, where the utility of a bundle is modeled in terms of weights assigned to coalitions of bundle attributes via the Choquet integral. This allows us to account for correlations between bundle attributes in an intuitive and principled way, capturing preferences that cannot be represented with standard solutions based on weighted sums.

A recommendation system can be defined as *environmentally aware* when it also considers the environmental footprint of its recommendations individually or as a whole to generate recommendations. Although sustainability is a quite new aspect in the IT sector and even less considered in the field of recommender systems, sustainable-aware systems have been proposed. For instance, a multi-stakeholder utility model is proposed for travel itinerary optimization (Merinov et al., 2022) and promoting the production of environmental-friendly products (Pachot et al., 2021). Tomkins et al. (2018) proposed a flexible probabilistic framework that uses domain knowledge to identify sustainable products and customers and uses these labels to predict customer purchases. We show how our formulation can be easily applied to compute an environmental friendliness score that depends on bundle attributes in a complex way, and select bundles of products that reduce the environmental impact.

#### 2.1.2 Choquet integral

Choquet integrals are sophisticated rank-dependent aggregators providing a fine control of interactions between any subset of criteria (Grabisch and Labreuche, 2008). The Choquet integral is an aggregation function defined with respect to a capacity (also called fuzzy measure).

Let X be the set of alternatives (items, products, candidates…) that need to be compared to make a decision. Any alternative x∈X is evaluated with respect to a set of *n* criteria denoted *N* = {1, …, *n*}, and is characterized by a performance vector (*x*_1_, …, *x*_*n*_); for all *i* ∈ *N*, *x*_*i*_ ∈ [0, 1] represents the utility of *x* with respect to the criterion *i*. For simplicity, *x* will indifferently denote the alternative or its performance vector. For any alternative x∈X, let (.) denote the permutation of {1, ⋯ , *n*} which sorts the components of *x* by increasing order, i.e., *x*_(*i*)_ ≤ *x*_(*i*+1)_ for *i* ∈ [[1, *n* − 1]]. Let *X*_(*i*)_ denote the subset of criteria with respect to which *x* has an utility greater or equal to *x*_(*i*)_, i.e., *X*_(*i*)_ = {(*i*), …, (*n*)}; note that *X*_(*i*+1)_ ⊆ *X*_(*i*)_ for all *i* ∈ [[1, *n*−1]] by definition. In the sequel, *X*_(*i*)_ will be referred to as the *i*^*th*^ *level set* of *x* and *Y*_(*i*)_ will denote the *i*^*th*^ *level set* of an alternative y∈X.

Let *μ* be a Choquet capacity, i.e., a set function defined on 2^*N*^ where *μ*(*A*) representing the weight attached to coalition *A*, for any *A* ⊆ *N*. A capacity must be such that

- *μ*(∅) = 0, *μ*(*N*) = 1 and
- *μ*(*A*) ≤ *μ*(*B*) for all *A* ⊆ *B* ⊆ *N* *(monotonicity)*.

Now, the Choquet integral is defined by:


$$\begin{array}{l} C_{\mu }\left(x\right)=x_{\left(1\right)}\mu \left(X_{\left(1\right)}\right)+\overset{n}{\underset{i=2}{\sum }}\left[x_{\left(i\right)}-x_{\left(i-1\right)}\right]\mu \left(X_{\left(i\right)}\right) \end{array}$$

Hence, an alternative *x* is at least as good as *y* whenever *C*_*μ*_(*x*) ≥ *C*_*μ*_(*y*). For example, consider a problem defined on 3 criteria {1, 2, 3} and *x* = (5, 3, 7) and *y* = (5, 6, 4) are two performance vectors. The computation of their Choquet value with the following capacity *μ* gives:

**Table:** 

	∅	{1 }	{2 }	{3 }	{1,2 }	{1,3 }	{2,3 }	{1, 2, 3 }
*μ*	0	0.1	0.2	0.4	0.3	0.8	0.6	1


$$\begin{array}{l} C_{\mu }\left(x\right)=3+\left(5-3\right)\mu \left(\left\{1,3\right\}\right)+\left(7-5\right)\mu \left(\left\{3\right\}\right)=\;5.4\\ C_{\mu }\left(y\right)=4+\left(5-4\right)\mu \left(\left\{1,2\right\}\right)+\left(6-5\right)\mu \left(\left\{2\right\}\right)=\;4.5 \end{array}$$

Hence, we have *C*_*μ*_(*x*) > *C*_*μ*_(*y*), meaning that *x* is strictly preferred to *y*. In multi-criteria decision-making, one needs to ensure that *C*_*μ*_(*x*) ≥ *C*_*μ*_(*y*) whenever *x* weakly Pareto-dominates *y* (i.e., *x*_*i*_ ≥ *y*_*i*_ for all *i* ∈ *N*). This property holds due to the monotonicity of *v* with respect to set inclusion.

An alternative method for computing the Choquet value makes use of Möbius masses. The Möbius masses associated with a capacity *μ* are such that $\mu \left(A\right)=\underset{B\subseteq A}{\sum }m\left(v\right)$.


(1) $$\begin{array}{l} C\left(x\right)=\underset{V\;\subseteq \;X}{\sum }m\left(V\right)\;\min\left(\left\{x\;|\;x\in V\right\}\right) \end{array}$$

The Choquet integral is quite general as an aggregation method, as it encompasses other aggregators as a special case. In particular, we emphasize two particular cases:

- A capacity is *additive* if, for all disjoint *A, B* ⊆ *N*, we have that *μ*(*A* ∪ *B*) = *μ*(*A*) + *μ*(*B*). If *μ* is additive, then the Choquet integral reduces to a weighted mean:
$$\begin{array}{l} C_{\mu }\left(x\right)=\underset{i\in N}{\sum }\mu \left(\left\{i\right\}\right)x_{i}. \end{array}$$
- A capacity is *symmetric* if, for any subsets *A, B*, |*A*| = |*B*| implies *μ*(*A*) = *μ*(*B*). If *μ* is symmetric, the Choquet integral reduces to the so-called *Ordered Weighted Average* (OWA) introduced by Yager (1988):
$$\begin{array}{l} C_{\mu }\left(x\right)=\underset{i\in N}{\sum }\left(\mu_{n-i+1}-\mu_{n-i}\right)f_{\sigma \left(i\right)} \end{array}$$
  with *μ*_*i*_ = *μ*(*A*), such that |*A*| = *i* and σ is defined as before.

#### 2.1.3 Preference elicitation

Preference elicitation is a crucial task in modern artificial intelligence. Learning or eliciting preferences means acquiring preference information in either direct or indirect way, from preference statements, critiques to examples, observations of user's clicking behavior, etc.

Modern approaches to preference elicitation often rely on interactive methods that incrementally acquire preferences by asking questions (also called queries) that are picked in an adaptive way, so as to improve the knowledge about the user's preferences. The emphasis is on providing recommendations of good quality with limited information about the user preferences; this stands in contrast to traditional elicitation techniques that need to fully determine a precise user model before making a decision.

From a high level point of view, many studies on systems for automated elicitation and recommendation adopt the following common scheme, where a “belief” is maintained about the user's preferences, represented by a utility function *u*, and the interaction proceeds as follows:

- Some initial user preferences are given, and the belief is initialized.
- **Repeat** until the belief meets some *termination condition*.

1. Ask user a query *q*
2. Observe the user response *r*
3. Update the belief given the answer *r* to the query *q*

- Recommend the item optimal according to the current belief.

During the above process, as more questions are answered, the acquired information allows the system to refine the belief with increasing precision. As the belief is refined, the system is able to make better and better recommendations; often, the system might be able to recommend the true best alternative (the alternative associated with maximum utility according to the user's utility function) even if the utility function is not known exactly.

To make this scheme concrete, it is necessary to specify how uncertainty in the preferences is represented. One popular approach assumes a Bayesian point of view: the belief is represented by a probability distribution on parameters of the utility function. This has the advantage of allowing to account for different choice models when answering queries (handling noisy information) and can exploit prior information. However, probabilistic reasoning can be computationally expensive and not apt to all circumstances.

Another family of approaches assume *strict uncertainty*, where the belief is represented by constraints on the parameters of the utility function, with no further quantification of how likely specific utility functions are. In this case, the recommender maintains an explicit representation of a set of feasible utility functions, represented compactly by constraints on parameters. With strict uncertainty, a principle criterion for decision-making is the *minimax regret* criterion. This is used to allow the recommender system to pick an alternative when preferences are only partially known. Let *u*(·;*w*) be the utility function parametrized by *w*, a vector of weights; in the setting of strict uncertainty, the exact value of the parameters *w* is not known precisely but it is known that it can initially take any value in an uncertainty set denoted *W*. Given a set of possible parameter values *W*, we define *max regret (MR)* as:


$$\begin{array}{l} \mathrm{MR}\left(x;W\right)=\underset{w\in W}{\max}u\left(y;w\right)-u\left(x;w\right). \end{array}$$

The max-regret of an alternative *x* is the maximum loss (in term of utility) that can be incurred by not choosing the (unknown) true optimal alternative. The *minimax regret (MMR)* is:


$$\begin{array}{l} \mathrm{MMR}\left(W\right)=\underset{x\in X}{\min}\mathrm{MR}\left(x;W\right)=\underset{x\in X}{\min}\underset{w\in W}{\max}u\left(y;w\right)-u\left(x;w\right). \end{array}$$

and the recommended alternative x^*^ (ties are broken in an arbitrary way) is


$$\begin{array}{l} x^{*}\in \arg\underset{x\in X}{\min}\mathrm{MR}\left(x;W\right). \end{array}$$

Recommending x^*^, the alternative associated with minimax regret, allows one to guarantee that the worst-case loss is minimized.

According to the interactive elicitation process, questions are asked to reduce the uncertainty with the respect to the user's preferences; note that, when more information is considered, the space *W* becomes smaller, and minimax regret cannot increase, and often decreases. As *termination condition*, we adopt the fact that minimax regret drops lower than a (small) threshold: by recommending x^*^, we ensure that the final recommendation is near-optimal.

Questions are chosen to be as informative as possible with respect to some criterion or with heuristics to reduce minimax regret as fast as possible. The process continues until the moment where there is enough information to make a recommendation with enough confidence. Different types of queries can be used in an incremental elicitation process; comparison queries, are often used. Other query types include choice queries (that extend comparison queries to sets of alternatives) and bound queries (asking to assign a bound on specific parameters).

Questions are asked according to *query selection strategies* that pick the next question to ask based on the current preference information. A popular method is the *current solution strategy* that asks the decision maker to compare the regret-optimal alternative with its adversary.

##### 2.1.3.1 Regret-based elicitation for Choquet

We now assume that the preferences of the user are modeled by a Choquet integral and see how minimax regret can be used to recommend an alternative with partially specified capacity weights and as well develop an interactive elicitation process that repeatedly asks the user questions to progressively decrease the uncertainty on the weights.

In the following, we briefly summarize the approach of Benabbou et al. (2017) by associating each possible set of criteria in 2^*N*^ with a lower and an upper bound: for each *A* ⊆ *N*, *l*_*A*_ ≤ *μ*(*A*) ≤ *μ*(*A*). Initially (before the start of the interaction), we set *l*_*A*_ = 0 for all ∅ ⊂ *A* ⊂ *N*; and *u*_*A*_ = 1 for all ∅ ⊂ *A* ⊆ *N*. Note that, as *μ*(∅) = 0 by definition, we have *l*_∅_ = *u*_∅_ = 0; similarly, *l*_*N*_ = *u*_*N*_ = 1 since *μ*(*N*) = 1.

In practice, the vector (*l*_*A*_, *u*_*A*_)_*A*⊆*N*_ constitutes a compact representation of all possible capacities consistent with the available preference information, indeed any possible.

As queries, we consider comparison queries between specific hypothetical alternatives: binary alternatives and constant alternatives.

*Binary alternatives* of type 1*A*0, where 1*A*0 is an alternative with a top performance on all criteria in *A* ⊂ *N* and a bottom one on all others. For example, 1*A*0 = (1, 0, 1, 0, 0) when *A* = {1, 3} and *n* = 5. A peculiar property is that *C*(1*A*0) = *v*(*A*), that is, the value of the Choquet integral of 1*A*0 is equal to the capacity value *μ*(*A*) of the set *A*.

*Constant alternatives* are alternatives with the same value in all components, as Λ = (λ, …, λ). It is remarkable that *C*_*μ*_(Λ) = λ; the Choquet integral of Λ = (λ, …, λ) is equal to λ.

Therefore, when the user specifies a preference, such as 1*A*0⪰Λ, this implies that


$$\begin{array}{l} \mu \left(A\right)=C_{\mu }\left(1A0\right)\geq C_{\mu }\left(\Lambda \right)=\lambda . \end{array}$$

This means that user's responses can be incorporated in our model by simply updating the lower and upper bounds of the set of criteria. If the user prefers 1*A*0 to Λ, then *l*_*A*_ is updated to a new value, *l*_*A*_: = max(*l*_*A*_, λ); moreover, the lower bounds of all supersets *A*′ ⊆ *N* of *A* have to be updated to satisfy monotonicity. If instead, Λ is preferred to 1*A*0, then *u*_*A*_: = min(*l*_*A*_, λ); the upper bounds $u_{A^{\prime }}$ of all subsets *A*′ of *A* have to be updated to satisfy monotonicity.

In any case, whatever the answer of the user, the set of possible capacities is compactly represented by the (updated) vector (*l*_*A*_, *u*_*A*_)_*A*⊆*N*_. As the elicitation proceeds, the users will be asked to compare different binary alternatives to different constant alternatives, acquiring several constraints on the capacity, that will result in repeated updates on the lower and upper bounds of the set of criteria. By representing the capacity in such a way, it is possible, in any step of the elicitation, to compute minimax regret in an efficient way using either a linear programming method or an iterative algorithm (Benabbou et al., 2017) by picking the best recommendation given the current knowledge about the capacity.

### 2.2 Motivating example

Consider the following straightforward example of bundle recommendation in an online marketplace of food products. The platform seeks to promote product bundles that include locally produced products while minimizing the environmental footprint of the bundle itself. This aims to attract customers who share an interest in environmental responsibility and to raise consciousness regarding a more sustainable method of acquiring products.

In terms of suggesting such products, the following criteria may be defined:

1. **Same warehouse**: this criterion determines whether food items in the bundle are stored in the same warehouse. Intuitively, shipping from a single warehouse minimizes transportation emissions, making the bundle more environmentally friendly.
2. **Low carbon footprint**: this metric allows quantifying the greenhouse gases emitted during the production and refining of the bundle's products. The underlying assumption is that eco-friendly foods have a smaller negative impact on the environment due to their greater sustainability.

Suppose there exist three bundles, *b*_1_, *b*_2_, and *b*_3_ with the following scores on the aforementioned criteria: *b*_1_ = (1, 0), *b*_2_ = (0, 1), and *b*_3_ = (0.55, 0.4). Here, a score of 1 indicates that the respective criterion has been completely satisfied (e.g., all products in the bundle are stored in the same warehouse), while an intermediate score indicates that the criterion is satisfied by only a fraction of the products. By using a simple weighted sum model, it seems appropriate to assign both criteria equal contribution to the aggregated score (e.g., *w*_1_ = *w*_2_ = 0.5), this results in the following scores: WS(*b*_1_) = WS(*b*_2_) = 0.5, WS(*b*_3_) = 0.475. Therefore, according to the weighted sum model, *b*_1_ and *b*_2_ should be preferred over *b*_3_.

However, neither *b*_1_ nor *b*_2_ satisfy the requirements that an e-commerce company may have in offering eco-friendly products. The reason for this is that if we supply products from multiple locations (*b*_2_), the bundle's environmental footprint will increase due to the additional transportation required, whereas if we recommend *b*_1_, we would reduce the number of shipments but recommend products that were potentially manufactured in an environmentally harmful manner.

As a consequence, the most sensible recommendation would be *b*_3_, since almost half of the products offered are manufactured in a sustainable manner (which lessens the impact that they have on the environment) and more than half of the products come from the same warehouse (implying that there will be fewer shipments than *b*_2_). By using the weighted sum, however, *b*_3_ cannot be offered since ∄(*w*_1_, *w*_2_) such that WS(*b*_3_) ≥ WS(*b*_1_), WS(*b*_2_). Let us assume that there exists *w* = (*w*1, *w*2) that satisfies the following linear system:


$$\{\begin{array}{l} 0.55w_{1}+0.4w_{2}\geq w_{1}\\ 0.55w_{1}+0.4w_{2}\geq w_{2}\\ w_{1}+w_{2}=1 \end{array}$$

We have decided to restrict the sum of the weights, setting it equal to 1; however, it is worth noting that the outcome may be trivially generalized to weights *w*_1_ + *w*_2_ = *k* for any *k* > 0.

From the third equation of the system,we get *w*_2_ = 1 − *w*_1_, which when replaced in the first two equations gives us:


$$\begin{array}{r} 0.55w_{1}+0.4\left(1-w_{1}\right)\geq w_{1}\\ 0.55w_{1}+0.4\left(1-w_{1}\right)\geq 1-w_{1} \end{array}$$

Solving these inequalities for w_1_ yields:


$$\begin{array}{r} w_{1}\leq 0.47058\\ w_{1}\geq 0.5217 \end{array}$$

Thus, in this example, we argue that the weighted sum yields erroneous recommendations. This is because when we are maximizing the bundle recommendation using the weighted sum, we implicitly assume independence between criteria, despite the possibility of synergy between them. In fact, in this example, there is a need to model an interaction between the criteria such that their combined effect is greater than the sum of their individual effects.

To adequately represent this synergy in a Choquet integral setting, the following capacities can be defined:

- *μ*({sameWarehouse}) = 0.1
- *μ*({lowCarbonFooprint}) = 0.1
- *μ*({sameWarehouse, lowCarbonFootprint}) = 1

A Choquet integral parameterized with these capacities allows the more eco-friendly bundle *b*_3_ to be proposed, as *C*(*b*_3_) = 0.415 ≥ *C*(*b*_1_) = *C*(*b*_2_) = 0.1.

### 2.3 Our approach

Given a set of *n* products $P=\left\{p_{1},p_{2},...,p_{n}\right\}$ and a reference product $p_{ref}\in P$ that the user is currently visualizing, our approach recommends a product bundle composed of *k* products $B=\left\{p_{1},p_{2},...,p_{k}\;|\;\forall ip_{i}\in P\right\}$ associated to the reference product *p*_*ref*_. Bundle recommendation can be framed as an optimization problem that aims to select an optimal set of items from a pool of candidates according to a given scoring function for the bundle (Shao et al., 2022).

**Figure 1:**
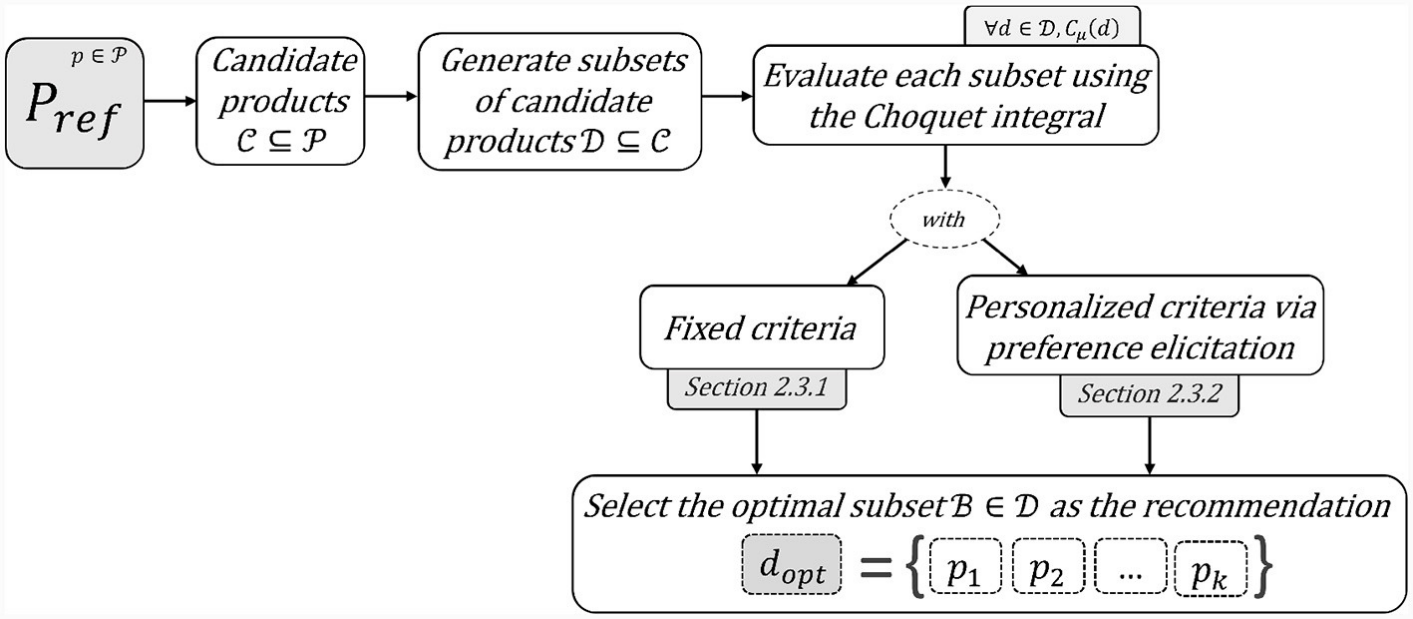
General framework for bundle recommendation.

Figure 1 presents a high-level overview of the proposed framework. After identifying a set of candidate products C for each product $p_{ref}\in P$, we evaluate each subset D⊆C using the Choquet integral *C*_*μ*_ as a scoring function. Section 2.3.1 describes the use of the function with fixed weight/capacity values *μ* for the bundle attributes. Whereas, Section 2.3.2 illustrates the elicitation of these values using preference elicitation. The recommended bundle B is finally elected for the product *p*_*ref*_ by selecting the product subset with the highest score, maximizing the explicated or learned criteria.

#### 2.3.1 Product-based bundle recommendation

First, a set of candidate items C⊆P should be identified by considering a subset of items that can be associated with the reference item $p_{ref}\in P$. Potential association criteria include shared attributes (e.g., same brand or vendor) or domain-dependent criteria (e.g., market segment or user preferences). In our case, we use the “*Production Area”* (*pa*) attribute to identify the subset of associated products. Accordingly, the subset of candidate products C is composed of products having the same production area as the reference product *p*_*ref*_. This product attribute has been chosen to enhance explainability as well as the territoriality of recommendations. The latter is also an important aspect since the considered e-commerce platform aims to promote product territoriality. Product territoriality is used, for instance, to generate territorial product bundles automatically as well as provide users with non-trivial explanations.

Once the set of associated products C⊆P has been identified as the candidate product set, an optimal subset of products B∈C should be selected from them. To achieve this objective, all possible subsets of products with cardinality less than a fixed maximum number *k* of elements (e.g., *k* = 4) are considered:


(2) $$\begin{array}{l} \forall \;D\subseteq C\;:\;1\leq |D|\leq k. \end{array}$$

For instance, assuming a user is visualizing the e-commerce page containing the product “Apple Cider Vinegar” (*p*_*ref*_), its production area (*pa*_*ref*_ = “Val di Non”) is used to retrieve other products produced in the same area (i.e., candidate products C⊆P). Afterwards, all possible subsets of this set of candidate products D⊆C are generated (Equation 2). All these product subsets are then assessed through some criteria which are evaluated using the Choquet integral as an aggregation function which computes a numerical score *C*_*μ*_ for each subset D⊆C. Finally, the optimal product bundle B∈D is selected as a bundle recommendation for $p_{ref}\in P$ by picking the product subset achieving the highest subset score:


$$\begin{array}{l} B:=d\in D\;|\;C_{\mu }\left(d\right)=\max\left(C_{\mu }\right) \end{array}$$

**Figure 2:**
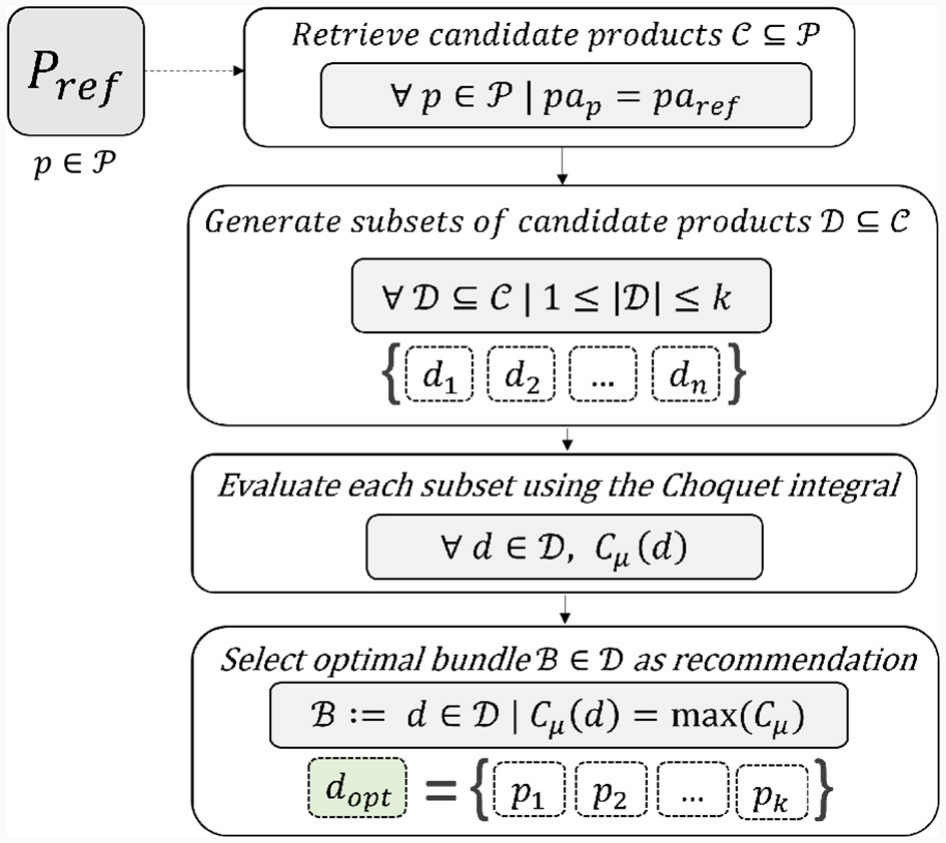
Our approach for bundle recommendation using the Choquet integral with fixed criteria.

Figure 2 provides a schematic summary of our proposed approach for the product-based bundle recommendation based on the use of the Choquet integral with fixed criteria.

#### 2.3.2 Preference elicitation with the Choquet integral

We elicit the preferences on the features of product bundles based on the regret-based interactive approach described in Section 2.1.3.1, by adopting the methodology by Benabbou et al. (2017). The main peculiarity of our framework is that we deal with bundles of products (sets of alternatives) rather than single alternatives.

The space of feasible parameters is:


$$\begin{array}{l} W={\left(l_{A},u_{A}\right)}_{A\subseteq N} \end{array}$$

Where *l*_*A*_ and *u*_*A*_ are the lower and upper bound of the capacity *μ*(*a*): *l*(*A*) ≤ *μ*(*A*) ≤ *u*(*A*).

The user is asked a series of questions comparing two hypothetical bundles. In accordance with the assumption of presenting the user with two sorts of options, namely those with constant utility (Λ = (λ, …, λ) ∈ [0, 1]^*n*^) and binary alternatives (e.g., 1*A*0 = (1, 0, 1, 0, 0) when *A* = {1, 3}), the technique involves identifying, for each *A* ⊆ *N*, the lambda value that minimizes the worst-case minimax regret to the greatest extent possible, which is defined as:


$$\begin{array}{c} \mathrm{WMMR}\left(\left(A,\lambda \right),W\right)\\ =\max\left\{\mathrm{MMR}\left(B,W_{\left(1A0,\Lambda \right)}\right),\mathrm{MMR}\left(B,W_{\left(\Lambda ,1A0\right)}\right)\right\} \end{array}$$

Where *W*_(1*A*0,Λ)_ is the set of possible parameter values with an added preference of 1*A*0 over Λ. After completing this phase, the subset A^*^ that optimally minimizes the WMMR is selected, and the user will thereafter be provided with the two choices generated by the pair $\left(A^{*},\lambda_{A^{*}}\right)$, which represents the most optimal query for the WMMR criterion.

**Figure 3:**
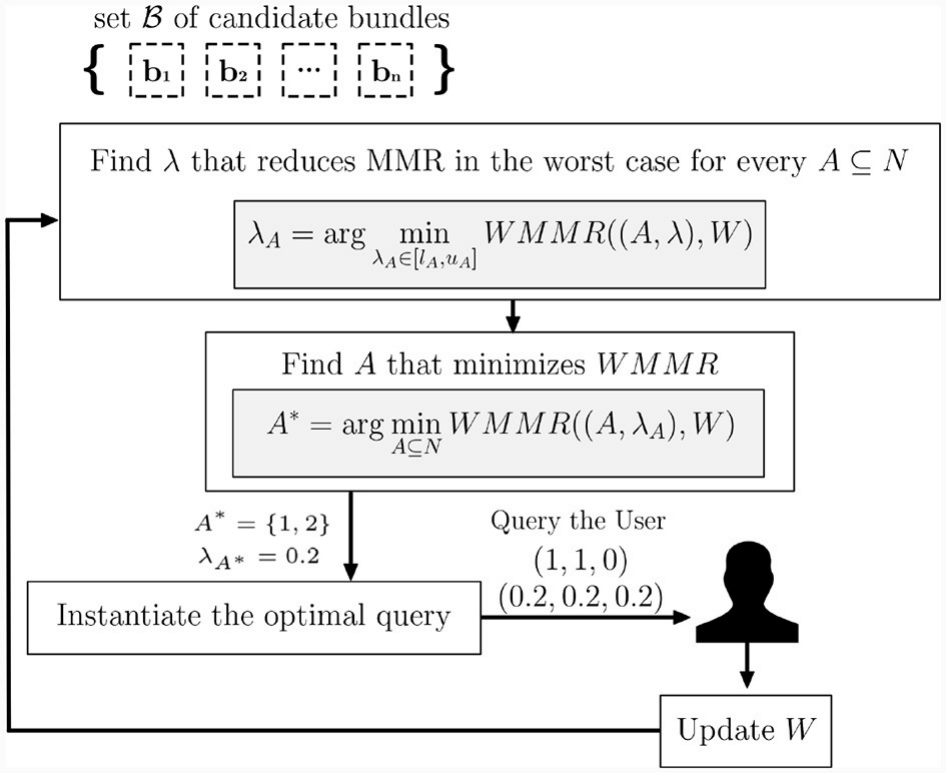
Diagram of the adopted elicitation process.

After receiving the user's choice, we proceed to update the parameter space in the manner presented in Section 2.1.3.1. For instance, as supposed in the illustration shown in Figure 3, let us assume that the algorithm asks the user to compare the constant utility alternative (0.2, 0.2, 0.2) and the binary alternative (1, 1, 0). Assume also that we are at the first step of the elicitation procedure; thus, the true capacity values fall inside the following interval [0, 1]. In the scenario, where the user expresses a preference for the constant alternative (0.2, 0.2, 0.2), the intervals involved would be reduced to:


$$\begin{array}{l} \mu \left(\left\{1,2\right\}\right)\in \left[0,0.2\right]\text{ and therefore }l_{1,2}=0,u_{1,2}=0.2\\ \mu \left(\left\{1\right\}\right)\in \left[0,0.2\right]\text{ and therefore }l_{1}=0,u_{1}=0.2\\ \mu \left(\left\{2\right\}\right)\in \left[0,0.2\right]\text{ and therefore }l_{2}=0,u_{2}=0.2 \end{array}$$

and the other capacity values are left unchanged. Alternatively, if the user prefers instead the binary alternative (1, 1, 0) to the constant (0.2, 0.2, 0.2), the following value is updated


$$\begin{array}{l} \mu \left(\left\{1,2\right\}\right)\in \left[0.2,1\right] \end{array}$$

Following this, we continue presenting queries to the user until the level of regret diminishes to zero, or until a certain termination criterion (e.g., the maximal number of questions is reached) is met.

#### 2.3.3 Explainability with the Choquet integral

Adopting the Möbius variant of the Choquet integral enables us to improve the interpretability of the aggregation process by considering the individual significance, or weight, of an attribute coalition, regardless of the importance of its sub-coalitions.

This formulation allows us to exploit this information to generate automatically an explanation for each subset of candidate products (D⊆C). First, relevant attributes are extrapolated for each subset D by exploiting the computation of the Choquet integral in its Möbius formulation (Equation 1) with a numerical threshold *k* (e.g., *k* = 0.1):


$$\begin{array}{l} \forall V\subseteq X\;|\;z\left(V\right)\geq k \end{array}$$

(3) $$\begin{array}{l} z\left(V\right):=m\left(V\right)\;\min\left(\left\{x\;|\;x\in V\right\}\right) \end{array}$$

**Figure 4:**
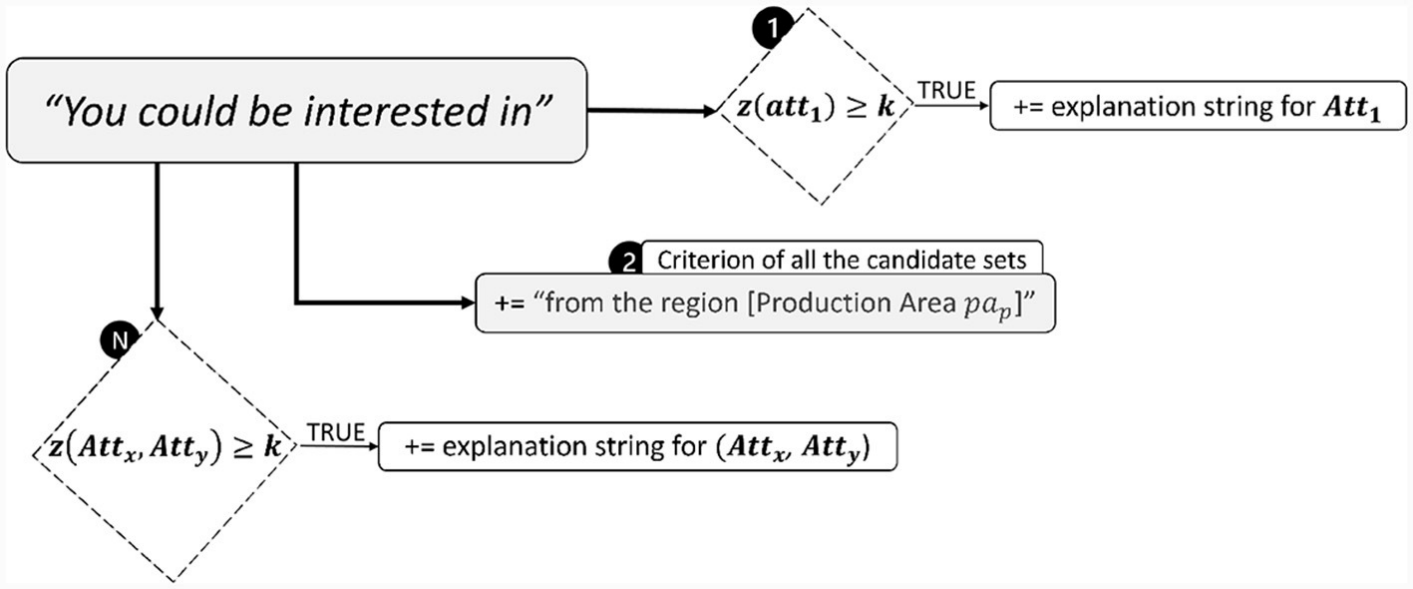
Sequential conditions to generate the bundle explanation text based on the relevant bundle attributes extrapolated from the computation of the Choquet integral.

The above formulation (Equation 3) allows us to jointly consider the importance [Möbius mass, m(V)] and the values themselves (bundle attribute values, x∈V) of each attribute subset V considered during the computation of the Choquet integral. Finally, the relevant attributes retrieved for each subset of candidate products D⊆C are exploited to generate an explanation text through a simple sequence of conditions as depicted in Figure 4.

#### 2.3.4 Scalability

Because users need time and effort to express their preferences, AI approaches to preference elicitation focus on the trade-off between decision accuracy and user effort (Peintner et al., 2008; Pigozzi et al., 2016). We argue that, in bundle recommendations, the size of a bundle will be limited because of the notion of *bounded rationality*, the idea that decision makers have limited cognitive resources and are more likely to make choices that are satisfying rather than optimal. When asking choice queries (among a set of alternatives, the user has to select the one she prefers), interactive elicitation methods focus on sets of cardinality up to five (Viappiani and Boutilier, 2020) (although an optimally rational decision maker will provide very informative feedback with larger sets).

The computation of minimax regret has quadratic cost in the worst case, and it is usually faster than the Bayesian approaches that require maintaining a probability distribution.

The bottleneck can be the computation of the next query to ask that, in principle, would require to consider all infinite possible questions to pick the one with the greatest regret reduction. By using the approach of Benabbou et al. (2017), we consider a limited set of potentially strong queries to decide the next one. Indeed, even though the generation of a single query can be computationally expensive, as the method explained in Section 2.3.2 implies a selection of A^*^ among 2^*N*^ − 2 subsets of *N*, our experimental evaluation shows that a handful of queries is sufficient to learn customized utility functions recommending optimal personalized bundles for a diverse set of user prototypes. Nevertheless, when *N* is significant, Benabbou et al. (2017) propose an heuristic that focuses on sets $A_{\left(x^{*},y^{*}\right)}=\left\{X_{\left(i\right)}^{*},i\in N\right\}⋃\left\{Y_{\left(i\right)}^{*},i\in N\right\}$, where x^*^ is the bundle that minimizes MMR, and y^*^ is an adversary choice; this results in a speedup in the execution time, as at most 2*N* − 1 sets are considered instead of 2^*N*^ − 2.

## 3 Results

Our experimental evaluation aims to study the effectiveness of the Choquet integral in modeling utility functions that depend on bundle attributes in non-trivial ways and the ability of our preference elicitation strategy to adapt the Choquet integral to specific users.

### 3.1 Recommending environmentally friendly bundles

In this section, we study the effectiveness of the Choquet integral in modeling the environmental friendliness of bundles on real data. We thus compare it to an alternative approach that simply models environmental friendliness as a weighted sum of bundle attributes. To make the comparison independent of the choice of the attribute weights, and show that the Choquet integral is *intrinsically* better, we perform a linear regression and learn the weights that maximize the environmental friendliness score of the resulting weighted sum. We perform an analysis to address the following research questions:

1. Does the Choquet integral generate different product bundles in comparison with a weighted sum?
2. Does the Choquet integral generate more environmentally friendly product bundles?
3. Does a recommender system using the Choquet integral recommend more environmentally friendly product bundles in practice?

#### 3.1.1 Experimental setting

**Figure 5:**
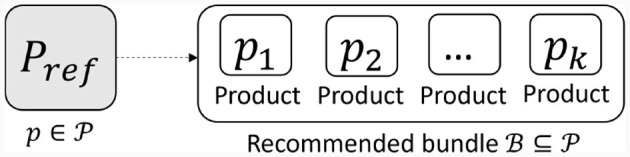
Diagram of the experimental setting for this use case. Given a reference product *p*_*ref*_, a bundle of products is recommended.

We consider an e-commerce platform from the Trentino region in North Italy that recommends local food products. The goal is to empower this platform with a bundle recommendation functionality that proposes a bundle of associated products on each product page. Given a set of *n* products $P=\left\{p_{1},p_{2},...,p_{n}\right\}$ and a reference product $p_{ref}\in P$; the proposed approach aims to suggest a product bundle composed of *k* products $B=\left\{p_{1},p_{2},...,p_{k}\;|\;p_{k}\in P\right\}$ associated with the reference product *p*_*ref*_ (Figure 5).

##### 3.1.1.1 Dataset

Each product $p_{i}\in P$ is characterized by *m* attributes $Y=\left\{y_{1},y_{2},...,y_{m}\right\}$. In our domain, the relevant attributes for identifying a product and computing the environmental friendliness of a candidate bundle containing it are: the *product name* (*y*_1_), the *production area* (*y*_2_), the *warehouse name* (*y*_3_), the *product weight* (*y*_4_), and *conservation method* (*y*_5_). For example, the product “Apple Cider Vinegar” is produced in Val di Non, a valley located in Trentino, and it is stored in the warehouse of the city of Trento; it has a weight equal to 700 grams and does not require refrigeration.

##### 3.1.1.2 Environmental friendliness score

Each subset of candidate products D⊆C is characterized by three attributes $X=\left\{x_{1},x_{2},x_{3}\right\}$ modeling the properties of the bundle in terms of the relationships between its items. The relevant attributes to characterize the environmental friendliness of a bundle are the following:

1. **Same warehouse (*x*_1_)**: the proportion of bundle products that are stored in the same warehouse. This criterion models the preference for product subsets with products from the same physical location, aiming to reduce the environmental footprint of shipping them by *minimizing the number of shipments*;
2. **Same conservation method (*x*_2_)**: the fraction of bundle products that have the same method of product conservation. This criterion favors bundles with products requiring the *same type of transport* (e.g., a truck with/without a refrigerated compartment) or the *same parcel type* (e.g., a regular or refrigerated parcel);
3. **Weight similarity (*x*_3_)**: a measure of the similarity between the product weights. This is computed as the ratio between the minimum and maximum weight of the products included in the bundle. As bundles having products with similar weights will ideally have a better *packaging*.

The values of these attributes are in the range 0–1 in which 0 represents the least preferred and worst scenario, while a value equal to 1 expresses the preferred and best scenario. The overall preferred case is thus a bundle that contains only products that are located in the same warehouse (*x*_1_ = 1), have the same conservation method (*x*_2_ = 1), and have identical weight (*x*_3_ = 1). Nevertheless, these criteria do not have the same importance for maximizing environmental friendliness (Equation 4): being located in the same warehouse (*x*_1_) is more important and preferred in comparison to having the same conservation method (*x*_2_), while bundling products with similar weights (*x*_3_) is the least important criterion among them.


(4) $$\begin{array}{l} x_{1}≻x_{2}≻x_{3} \end{array}$$

Furthermore, to formalize the notion of a product bundle's environmental friendliness, it is essential to consider certain attribute synergies. For example, the joint importance of being located in the same warehouse (*x*_1_) and having the same conservation method (*x*_2_) is higher than their individual contributions:


$$\begin{array}{l} \left(x_{1},x_{2}\right)≻x_{1}≻\;x_{2} \end{array}$$

The Choquet integral is used to evaluate each candidate bundle D⊆C according to these three criteria. This aggregation function is parameterized by a capacity *μ* which represents the importance/weight of each criterion in the aggregation score (Section 2.1.2). As illustrated in the motivating example (Section 2.2), it has the advantage over a standard weighted sum of accounting for the interactions among criteria while keeping, as much as possible, the interpretability of linear models (Bresson, 2020). The capacity values *μ* indicate the importance of each (coalition of) attribute, enabling us to assess the environmental friendliness of a product bundle by weighting the individual and joint contribution of these bundle attributes.

Accordingly, the capacities for the attributes sameConservation (*x*_2_) and similarWeight (*x*_3_) have been set to zero due to their lack of individual utility to asses the environmental friendliness of a product bundle (Table 1). It is reasonable to attribute specific importance to the coalition of sameWarehouse (*x*_1_) and sameConservation (*x*_2_), the joint impact of the former with the attribute similarWeight (*x*_3_) as well as the joint importance of all of them (Table 1). This is justified by the synergistic relationships observed in these coalitions, underscoring their relevance in the assessment of the environmental friendliness of a bundle. Nevertheless, we contend that assigning importance to the coalition of sameConservation (*x*_2_) and similarWeight (*x*_3_) is not advisable, since the environmental friendliness of a bundle fulfilling this coalition but not satisfying sameWarehouse (*x*_1_) should not be rewarded.

**Table 1:** The capacity values *μ* adopted to formalize the concept of environmental friendliness of a bundle of food products, considering the three bundle attributes and their synergies.

**Bundle attribute**	**Capacity value**
*x*_3_	0
*x*_2_	0
*x*_1_	0.25
(*x*_2_, *x*_3_)	0
(*x*_1_, *x*_3_)	0.5
(*x*_1_, *x*_2_)	0.75
(*x*_1_, *x*_2_, *x*_3_)	1

Each product subset D⊆C is accordingly evaluated through the Choquet integral in its Möbius variant (*C*_*μ*_, Equation 1), which aggregates all the subset attribute values X while considering the importance of these attributes and their synergies through the capacity values *μ* (Table 1). Finally, the subset with the highest aggregate score is picked as recommended bundle B∈D for the reference product *p*_*ref*_.

#### 3.1.2 Experimental results

We conduct similarity comparisons between product bundles selected, and thus recommended, using two different aggregating functions: the Choquet integral (our approach) and the weighted sum (baseline). We first randomly discarded a subset of products assuming them to be unavailable and then selected the higher-scoring bundles according to the two aggregating functions ($B_{chq},B_{ws}\in D$). We repeated the procedure 1,000 times and reported average results for each production area.

**Figure 6:**
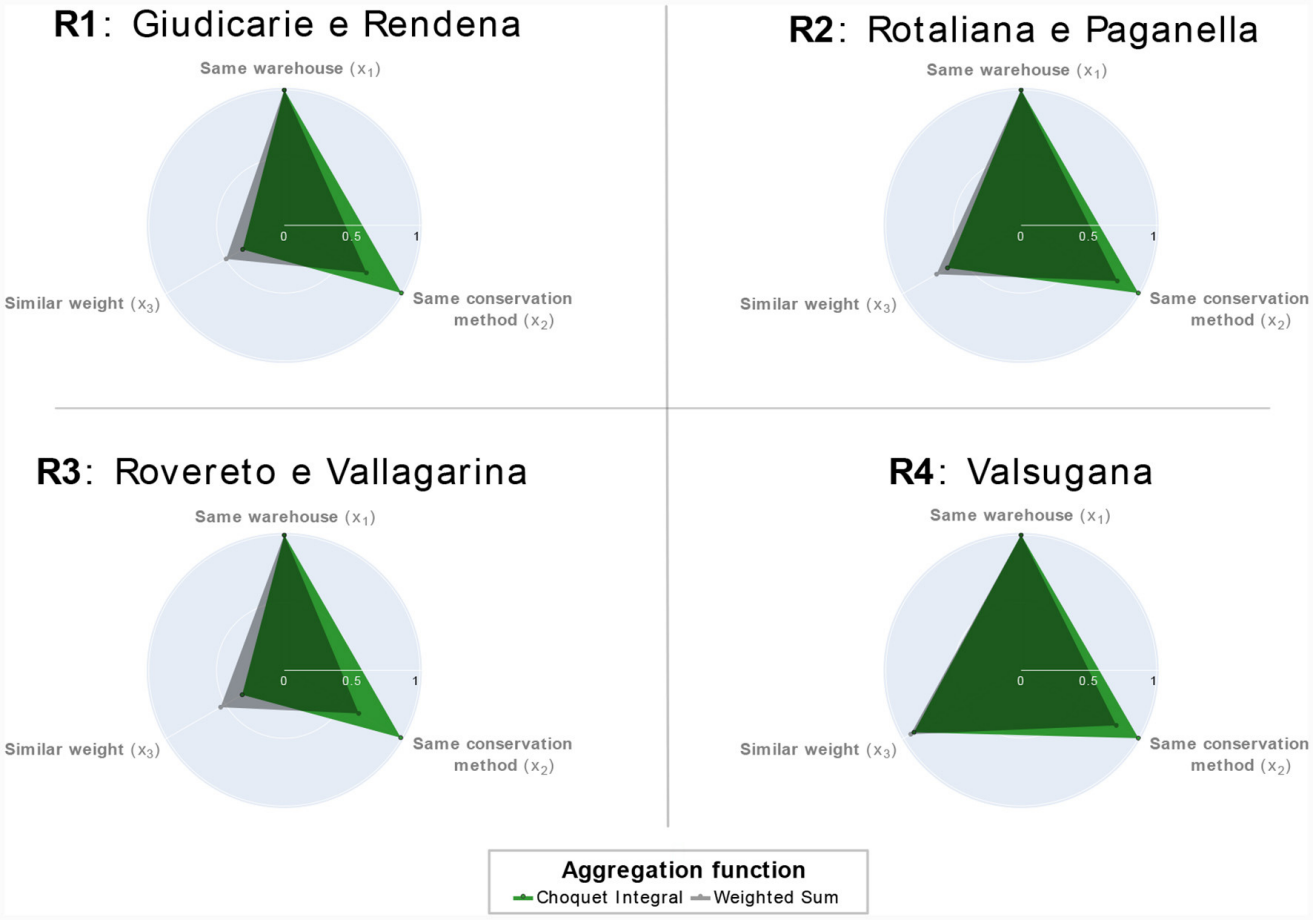
Bundle attributes of the product bundle selected by the two aggregating functions across all the simulations.

We first evaluated the impact of these aggregation functions on the recommendation process and their implications on the environmental friendliness of the selected bundles. The radar plots in Figure 6 visually exhibit the average environmental friendliness of the product bundles selected by the two aggregating functions across different production areas. This analysis demonstrates that adopting the Choquet integral allows us to select product bundles that are environmentally friendlier across all the considered production areas. It effectively captures the synergy between *x*_1_ and *x*_2_ while assigning less importance to the criterion *x*_3_. This discriminating behavior was not observed with the weighted sum, as remarked in the two left radar plots of Figure 6. Thus, employing the Choquet integral aids in effectively satisfying the attribute preferences and interdependencies described in Section 3.1.1.2. The Choquet integral consequently played an important role in formalizing the concept of environmental friendliness of a product bundle and accordingly selecting bundles with the highest environmental friendliness.

The second research question in our evaluation focuses on assessing the ability of a weighted sum model to approximate the environmental scores calculated through the Choquet integral. We employed linear regression to identify its optimal weights, aiming to linearly approximate the Choquet integral. These optimal weights are estimated by solving:


(5) $$\begin{array}{l} \begin{array}{l} \underset{w_{1},w_{2},w_{3}}{\min}\overset{n}{\underset{i=1}{\sum }}{\left(c_{i}-\left(w_{1}\cdot x_{1}+w_{2}\cdot x_{2}+w_{3}\cdot x_{3}\right)\right)}^{2}\\ \text{ s.t.}\;w_{1},w_{2},w_{3}\geq 0\\ \;\overset{n}{\underset{i=1}{\sum }}w_{i}=1\\ \;\text{solution found:}ŵ\approx \left(0.87,\;0,\;0.13\right) \end{array} \end{array}$$

**Figure 7:**
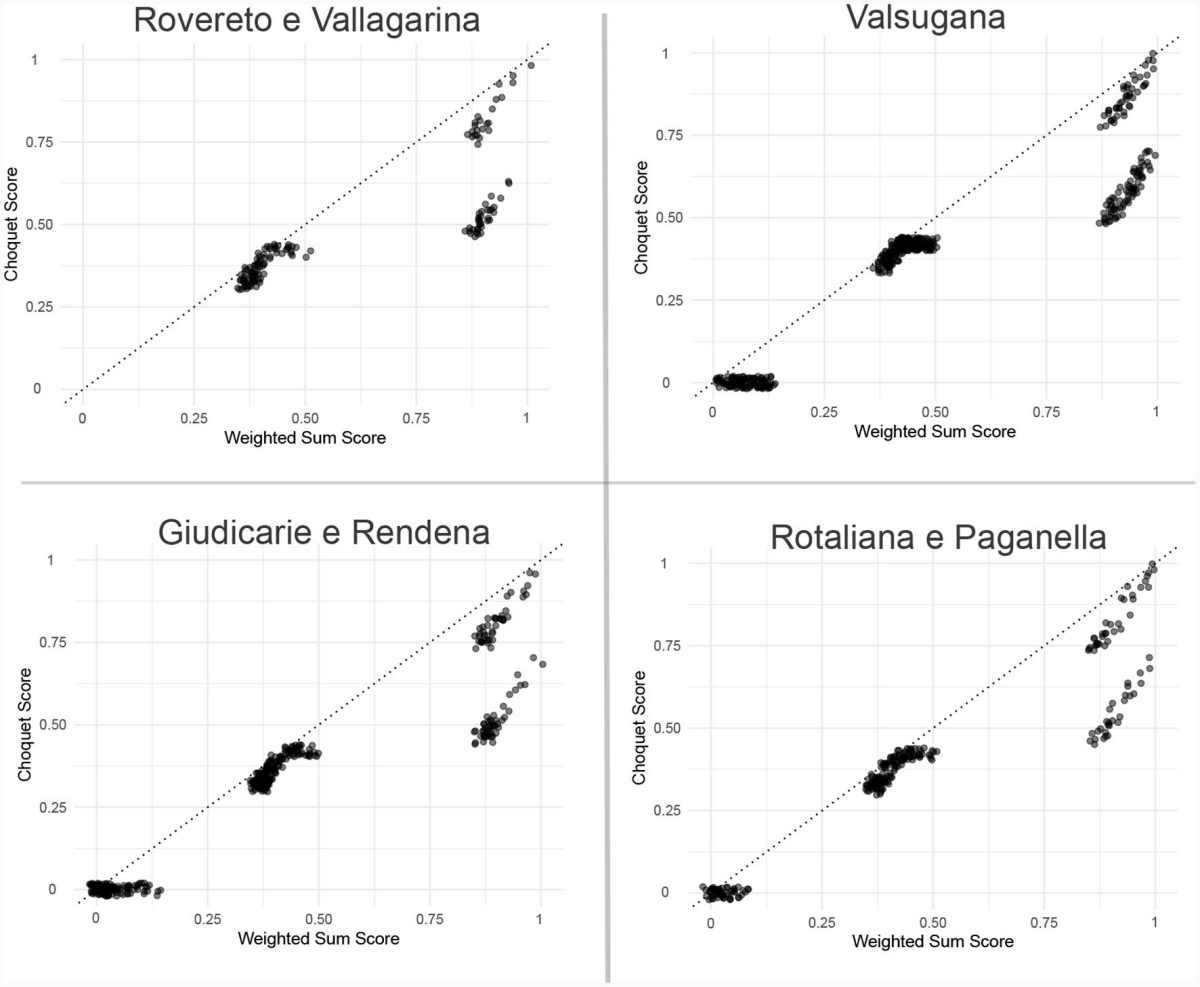
Choquet scores of the bundles and their corresponding predicted scores. To better view the data, jittering was applied.

Upon parameter estimation, a comparison was made between the Choquet scores of the bundles and their corresponding predicted scores. Figure 7 suggests the existence of bundles satisfying extensively all criteria, as observable in the top right corners of all the scatter plots (≈1). Although these observations have highly accurate predictions, there is another cluster of data points with similar weighted-sum scores but a lower Choquet value.

This finding can be explained by the attribute values and how the linear model ranks them. Specifically, although sameWarehouse and similarWeight exhibit a high degree of similarity between the two groups, there exists a disparity in the value of sameConservation (1 as opposed to 0.42) linked to the observations. This discrepancy accounts for the difference in the Choquet score. Furthermore, when combined with the fact that the linear model assigns a weight of zero to sameConservation (*w*_2_ = 0, Equation 5), it can be concluded that the model wrongly treats these two groups of bundles equivalently, thus failing to properly represent the non-linear behavior of the Choquet score. In addition, a residual analysis reveals observable patterns that suggest the insufficient validity of one of the assumptions underlying linear regression. In linear regression, it is expected that the residuals follow a random distribution centered around zero, with the absence of any discernible systematic patterns. Nevertheless, this is not the case.

**Figure 8:**
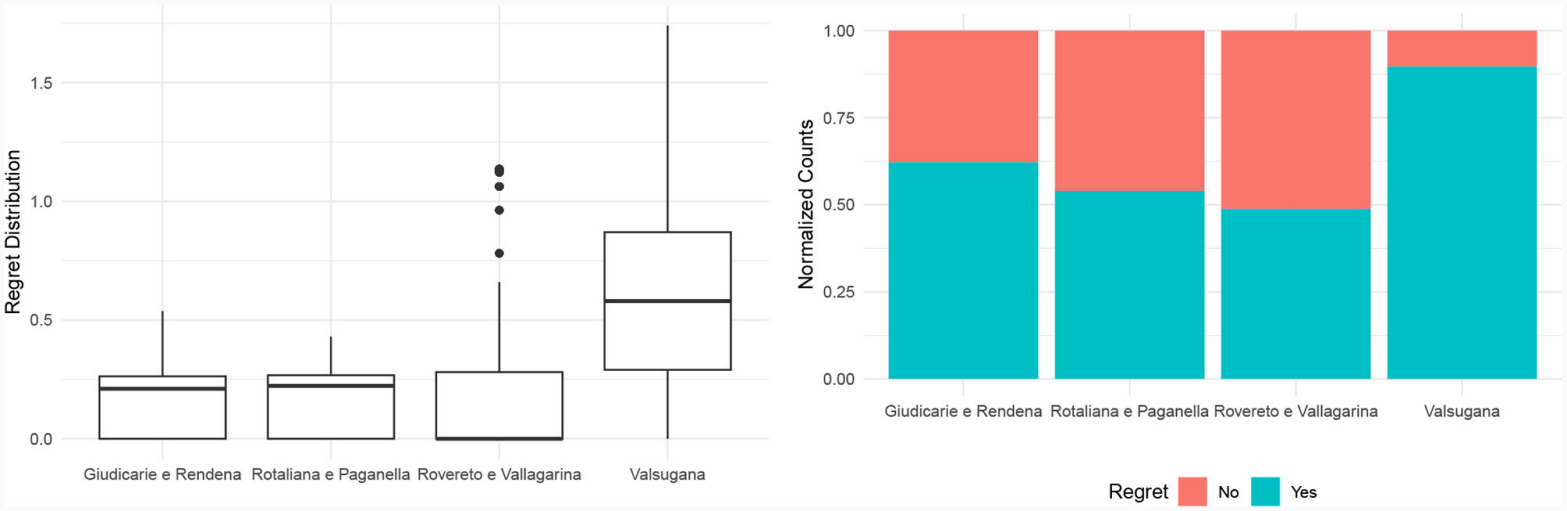
Regret distribution and frequency by production areas.

Hence, we claim that the weighted-sum model, despite parameter optimization, fails to effectively formalize the concept of environmental friendliness of a product bundle. Accordingly, our third and last research question for this evaluation introduces the concept of regret as the difference between the environmental friendliness score of two product bundles, respectively, selected by the Choquet integral and the weighted sum. To avoid trivial solutions, we focused on a setting where no bundle can jointly satisfy all criteria, and a trade-off has to be made to achieve optimal recommendations. We conducted 1,000 simulations where we randomly removed a fraction of items considering them as unavailable. For each simulation and production area, we extracted the bundles having the highest score according to the Choquet integral and the weighted sum, respectively. We then computed the regret of the weighted sum recommendation as the difference in environmental friendliness score between the two bundles. Figure 8 exhibits the distribution of the regret values across all the production areas, aggregating the one thousand simulations with random unavailable products. The limited availability of certain products has resulted in instances of regret in the recommendations, especially for the Valsugana production area. Figure 8 also provides a clearer understanding of the frequency by depicting the proportion of recommendations with and without regret. In all production areas, it is noticeable that the weighted-sum model often overlooked the optimal bundle that was available, in favor of a less sustainable alternative. In certain regions, this phenomenon occurs in more than half of all cases. Therefore, the linear model frequently yields inaccurate recommendations, even when applied to real-world scenarios where specific products are unavailable.

### 3.2 Recommending personalized bundles

In this section, we show how the preference elicitation strategy described in Section 2.3.2 allows to effectively adapt the bundle utility function to the characteristics of some prototypical users and quickly manages to recommend optimal bundles.

#### 3.2.1 Experimental setting

In this experimental setting, we propose an approach to generate product bundles personalized for different types of e-commerce customers (i.e., personas). The objective is to develop a recommendation system capable of recommending product bundles based on users' individual preferences learned via preference elicitation.

We first define a set of personas, consisting of prototypical representations of users who would engage in the purchase of food goods via an e-commerce platform. For each persona, we then identify a set of product attributes that could be relevant to their purchasing goals such as the absence of sugar for customers seeking healthy products (i.e., sugar-free products). Afterwards, we generate synthetic product data by adding these relevant attributes to the original dataset of food products.

##### 3.2.1.1 Dataset

Synthetic data for several new product-related attributes is generated by a two-step process. Initially, for each new attribute, a probability distribution is defined by following the sampling of the attribute's value. The majority of the variables are binary indicators, with their probability distributions specified in Supplementary Table S1, while some attributes have continuous or categorical values (Supplementary Figure S1). After generating the new attributes, potential bundles are generated by aggregating collections of up to five products. A complete list of bundle attributes is shown in the Supplementary material S2. The majority of bundle attributes quantifies the proportion of items inside the bundle that have a certain characteristic. The remaining ones are binary variables indicating whether *all* products of the bundle have a certain property, such as *Same Production Region, Same Vendor*, or *Eco-friendly Conservation Method*.

##### 3.2.1.2 Customer personas

We define three different types of customers, representing polarized e-commerce users who want to achieve specific purchasing goals:

1. **Net-zero persona**: A customer who would like to minimize the environmental footprint of their purchases regardless of potential drawbacks such as costlier products. This persona wants to minimize the life-cycle footprint of its purchased goods, from production to shipment. A bundle should include *organic* products (*z*_1_) from the *same warehouse* (*x*_1_), with an *eco-friendly conservation method* (*z*_2_, i.e., not refrigerated) and *similar weights* (*x*_3_). In addition, the products should have *recyclable* (*z*_3_) or *compostable packaging* (*z*_4_), and the producer should preferably have *environmental certificates* (*z*_5_, e.g., use of green energy). An example of attribute synergy for minimizing the overall environmental footprint of a bundle is the joint relevance of products with compostable packaging (*z*_4_), an eco-friendly conservation method (*z*_2_) and stored in the same warehouse (*x*_1_).
2. **Healthy persona**: A customer seeking healthy food products which should be *organic* (*z*_1_), *sugar-free* (*h*_1_), and with *low trans fat* (*h*_2_). Furthermore, the *absence of additives* (*h*_3_) and *sweeteners* (*h*_4_) should be positively considered. Finally, bundles of products with *low salt* (*h*_5_) should be rewarded. An example of attribute synergy is the joint absence of sugar (*h*_1_) and sweeteners (*h*_4_) in food products.
3. **Serendipitous persona**: A customer who would like to discover new products and tastes. An e-commerce platform should promote a bundle of *diversified products* (*s*_1_), with novelties for the user in terms of *new products or tastes* (*s*_2_) or *food categories* (*s*_3_). Alongside product novelty and diversity, *popular products* (*s*_4_) should be rewarded when recommending a bundle to a serendipitous customer. A potential attribute synergy arises from the combined impact of including new products for the user (*s*_2_) and a varied assortment of products (*s*_1_).
4. **Local SME-focused persona**: A customer who would like to support small- and medium-sized enterprises (SMEs) and the economy of her/his region. The platform should promote products from the *same region of the user* (*l*_1_) or offered by a *same-region vendor* (*l*_2_). Additionally, *homemade* (*l*_3_) or *fresh* products (*l*_4_) should be promoted. Furthermore, products with *ethical work certifications* (*l*_5_, e.g., SA8000 standard) or *supply chain traceability* (*l*_6_) should be rewarded. An example of attribute synergy is to reward products from the same region of the user (*l*_1_) and offered by a local vendor (*l*_2_).
5. **Ethical persona**: A customer who would like to purchase ethical products considering the entire product life cycle from farming to distribution. The platform should promote bundles of products with an *ethical work certification* (*l*_5_) and a *traceability of the supply chain* (*l*_6_). In addition, *organic* and *homemade products* (*z*_1_ and *l*_3_) should be rewarded. Finally, products with compostable packaging (*z*_4_) could enhance the customer's satisfaction. A potential attribute synergy arises from the joint contribution of *l*_5_, *l*_6_, and *z*_1_.

Supplementary material S3 presents all the capacity values and the attribute coalitions for each persona.

#### 3.2.2 Experimental results

We evaluated the effectiveness of the preference elicitation strategy presented in Section 2.3.2 by comparing it with a baseline approach where queries are selected at random. A random query is generated by first randomly choosing a subset *A* ⊂ *N*, and then sampling the lambda value λ_*A*_ from a uniform distribution $U\left(l_{A},u_{A}\right)$.

**Figure 9:**
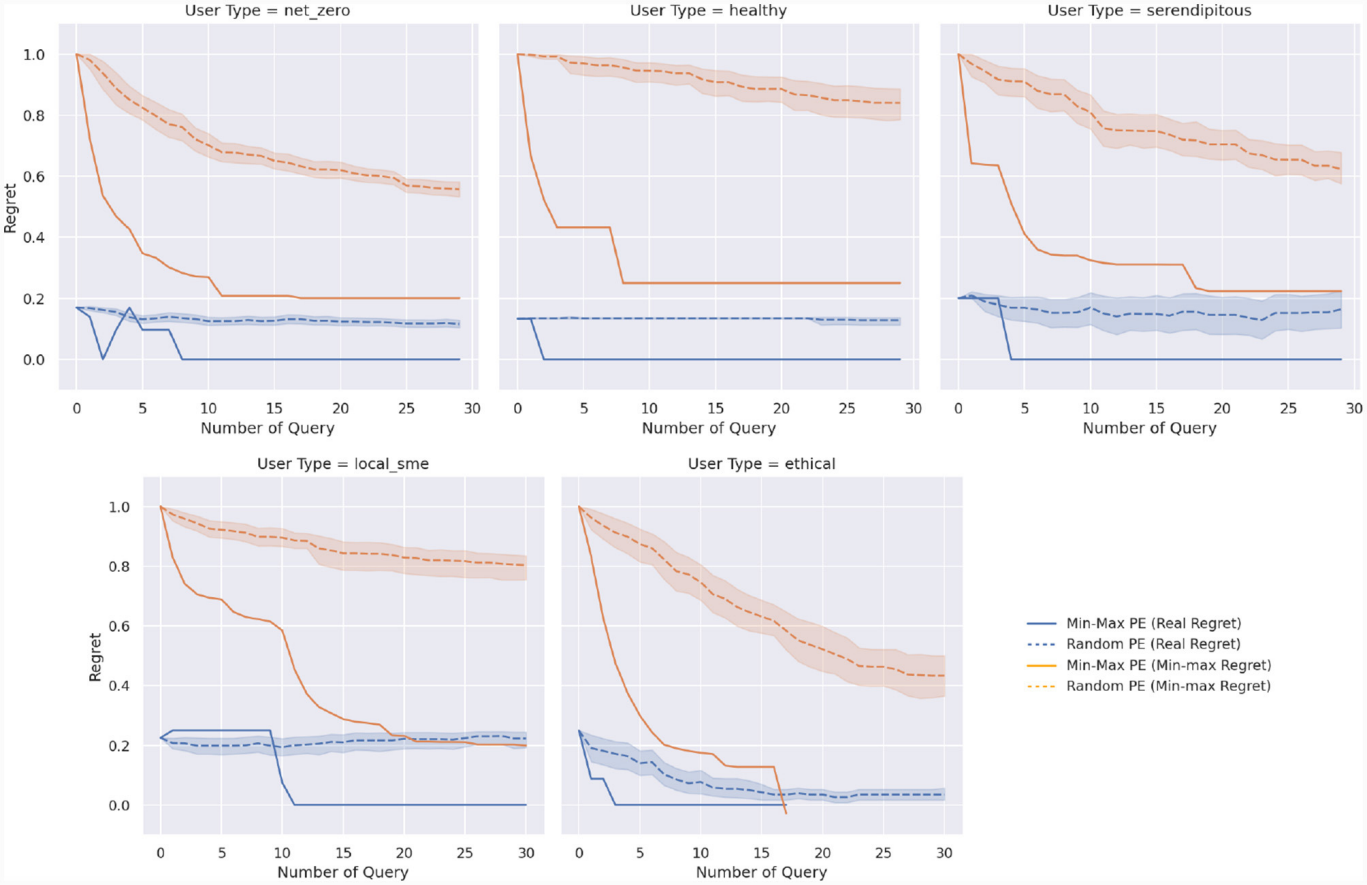
Elicitation of the preferences for the different types of customers for an increasing number of queries. Solid curves indicate results using the min-max regret preference elicitation strategy, while dashed curves report results with a random preference elicitation strategy. Blue curves report results in terms of real regret, while orange curves report min-max regret values.

Results are shown in Figure 9, with each graph focusing on a different persona. Orange curves report minimax regret as a function of the number of queries, while blue curves report the true regret, with a value of zero indicating the recommendation of an optimal bundle. Solid curves show the results of the preference elicitation strategy based on minimizing WMMR, while dashed curves indicate the random strategy, with the shadowed area representing a 95% confidence interval computed over 20 runs with different random seeds.

By looking at the orange curves, the effectiveness of the MMR-based preference elicitation strategy is apparent. Between one and five queries, depending on the persona, are sufficient to achieve a minimax regret that is comparable to 30 random queries. When considering the actual regret, it becomes evident that this strategy also succeeds in minimizing the regret of the final recommendation. Indeed, with a handful of queries (two to 11 depending on the persona), our approach manages to recommend an optimal, zero-regret bundle to each user. Conversely, the random strategy achieves a negligible reduction in regret, regardless of the number of queries being made.

**Figure 10:**
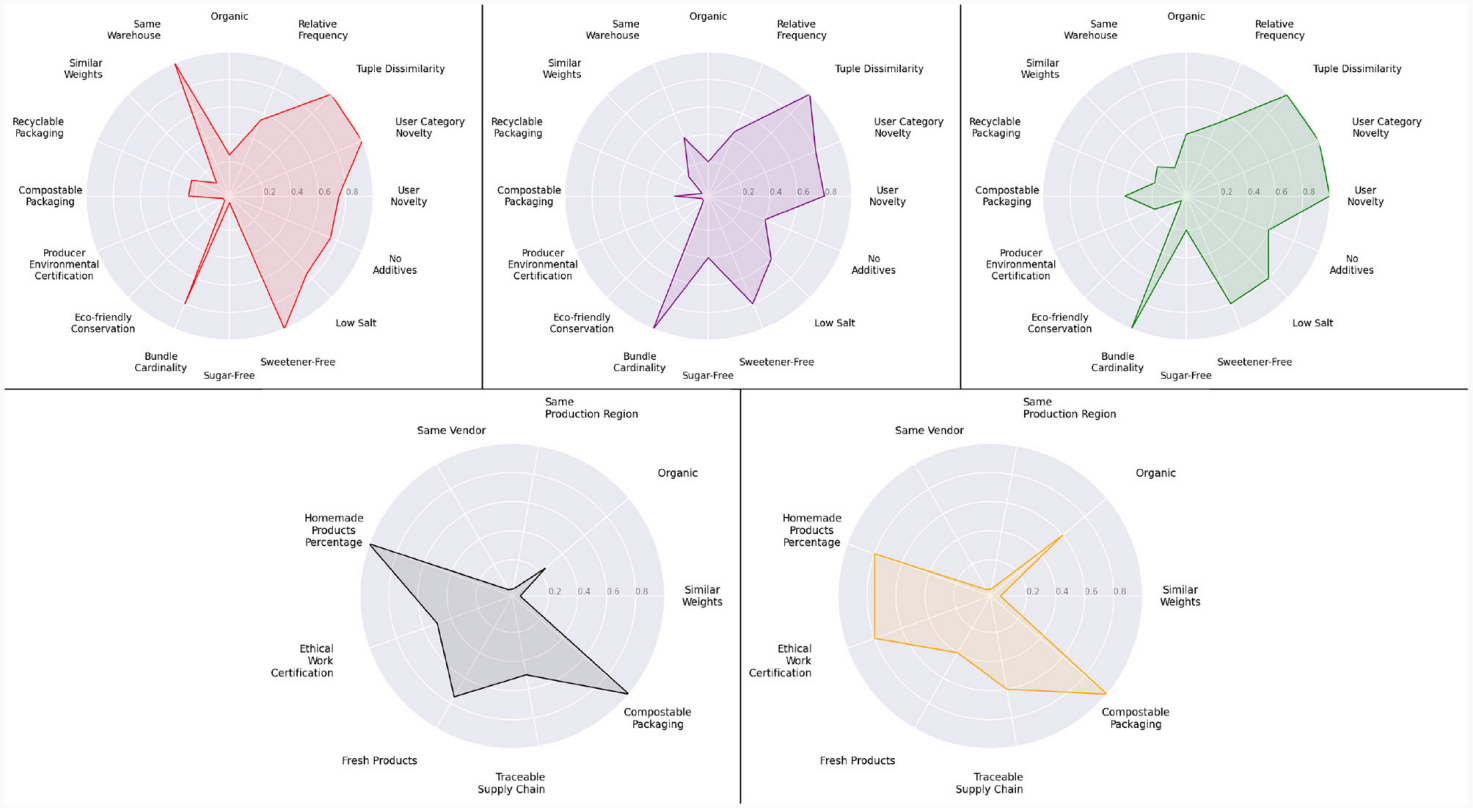
Performances of the suggested bundle for the net-zero persona (red), healthy persona (purple), serendipitous persona (green), local SME-focused persona (black), and ethical persona (orange). For readability purposes, the attributes of the illustrations for the local SME-focused persona and the ethical persona differ from those of the other personas.

Following the conclusion of the preference elicitation process, a visual inspection was conducted on the proposed bundles for each persona. As can be seen in Figure 10, each user is eventually presented with a distinct selection of food products. For the net-zero persona, the size of the bundle is reduced (BundleCardinality = 0.8) to guarantee that all products inside the bundle are located in the same warehouse (sameWarehouse = 1.0), hence minimizing the number of shipments; this is further supported by examining Table 2, which provides a comprehensive breakdown of the characteristics of the products comprising the bundle. The healthy individual, conversely, places greater emphasis on the attributes of low sodium, sugar-free, additive-free, and sweetener-free products (Table 3). Furthermore, a product bundle with maximal diversity and novelty is recommended for the serendipitous persona. Finally, bundles that prioritize SMEs items and ethical consumerism are suggested for the local SME-focused and ethical persona, respectively.

**Table 2:** Product attributes of the products recommended to the net-zero persona.

	**Product bundle proposed to the** ***net-zero persona***			
	**Trota salmonata affumicata affettata**	**Prosciutto cotto al vapore**	**Alperbit**	**Confettura extra di fragole**
Warehouse ID	0	0	0	0
Refrigerated	No	Yes	No	Yes
Recyclable package	No	Yes	No	No
Compostable package	No	Yes	No	No
Organic	No	No	No	Yes
Environmental certificate	No	No	No	No

**Table 3:** Product attributes of the products recommended to the healthy persona.

	**Product bundle proposed to the** ***healthy persona***				
	**Trentina Barrique**	**Pancetta cotta arrotolata**	**Alperbit**	**Primiero Fresco**	**Confettura extra di fragole**
No sweetener	No	Yes	Yes	Yes	Yes
Low salt	Yes	Yes	No	Yes	No
Sugar-free	Yes	No	No	No	Yes
No additives	No	Yes	Yes	No	No
Organic	No	No	No	No	Yes
Low trans fat	No	No	No	No	No

## 4 Conclusion

In this study, we showed how adopting the Choquet integral in a bundle recommendation systems allows us to naturally account for synergies among coalitions of attributes in modeling bundle utility functions. This can help formalize non-trivial concepts such as environmental friendliness and healthiness of bundles of food products. Recommending product bundles based on non-trivial concepts can provide concrete benefits for both the e-commerce platform and its customers. For instance, constructing environmentally friendly product bundles can help the e-commerce platform reduce its overall environmental footprint as well as improve operational efficiency. On the other hand, customers can visualize and purchase product bundles based on their high-level preferences (e.g., healthiness) rather than single-attribute preferences (e.g., sugar-free). Additionally, empowering the recommender systems with preference elicitation strategies allows to personalize bundle utility functions, by adapting the weights of the coalition of attributes to account for user feedback.

Finally, a relevant feature of the Choquet integral is its intrinsic explainability. When formalized in terms of Möbius masses, the weights of coalitions of attributes can be seen as indicators of their relevance in shaping the utility function, independently of the importance of their sub-coalitions.

## Data availability statement

The raw data supporting the conclusions of this article will be made available by the authors, without undue reservation.

## Author contributions

ER: Writing—original draft, Writing—review & editing. MB: Methodology, Writing—original draft, Writing—review & editing. PV: Methodology, Supervision, Writing—original draft, Writing—review & editing. AP: Methodology, Supervision, Writing—original draft, Writing—review & editing.
