# Supplementary material for: Personalized bundle recommendation using preference elicitation and the Choquet integral
Source: Front Artif Intell. 2024 Feb 14;7:1346684. doi: 10.3389/frai.2024.1346684 (PMC10899676; doi:10.3389/frai.2024.1346684)
Supplement: Supplementary file 1 [file Data_Sheet_1.pdf]

Supplementary Material

1 GENERATION OF THE SYNTHETIC DATA

**Binary Product Attributes**	$\mathbb{P}(x = 0)$	$\mathbb{P}(x = 1)$
Organic	0.8	0.2
Recyclable Product Packaging	0.8	0.2
Compostable Product Packaging	0.9	0.1
Producer Environmental Certificate	0.95	0.05
Sugar Free	0.8	0.2
Sweetener free	0.2	0.8
Low Trans Fat	0.6	0.4
Low Salt	0.3	0.7
No Additives	0.4	0.6
Bought by User	0.8	0.2
Is Category Novelty	0.1	0.9
Homemade	0.8	0.2
Ethical Work Certification	0.95	0.05

Table S1. Product attributes and their relative frequencies used to generate synthetic data.

**Buyers Percentage**	
**Value**	**Probability**
0	0.1
0.1	0.15
0.2	0.15
0.3	0.1
0.4	0.05
0.5	0.05
0.6	0.1
0.7	0.05
0.8	0.05
0.9	0.1
1	0.1

**Vendor**	
**Value**	**Probability**
A	0.1
B	0.3
C	0.2
D	0.3
E	0.05
F	0.05

Figure S1. Percentages of the buyer and vendor probability for synthetic data.

2 EXISTING AND SIMULATED ATTRIBUTES

Attribute
Bundle Composition
Same Conservation
Same Warehouse
Similar Weights
Bundle Cardinality
Items Similarity
Eco-friendly Conservation Method

Attribute
Organic (%)
Recyclable Product Packaging (%)
Compostable Product Packaging (%)
Producer Environmental Certificate (%)
Sugar-free (%)
Sweetener free (%)
Low Trans Fat (%)
Low Salt (%)
No Additives (%)
User Novelty (%)
User Category Novelty (%)
Homemade Products (%)
Global Relative Frequency (%)
Same Production Region (0/1)
Same Vendor (0/1)

Figure S2. List of the bundle attributes simulated (right) and the original ones (left).

3 CAPACITY VALUES FOR THE DIFFERENT PERSONAS

Bundle attribute	Capacity value
producerEnvCertificates (z_5)	0
similarWeights (x_3)	0
ecoConservationMethod (z_2)	0.05
recyclablePackaging (z_3)	0.05
compostablePackaging (z_4)	0.1
organicProducts (z_1)	0.1
bundleCardinality (z_6)	0.1
sameWarehouse (x_1)	0.2

Bundle attribute	Capacity value
(x_1, z_2)	0.3
(x_1, z_1)	0.4
(x_1, z_3)	0.3
(x_1, z_4)	0.4
(x_1, z_2, z_4)	0.5
(x_1, z_1, z_2, z_3)	0.6
$(x_1, z_1, z_2, z_3, z_4)$	0.7
$(x_1, z_1, z_2, z_3, z_4, z_6)$	0.8
$(x_1, z_1, z_2, z_3, z_4, z_5, z_6)$	0.9
$(x_1, z_1, z_2, z_3, z_4, z_5, z_6, x_3)$	1

Figure S3. The capacity values μ adopted to formalize the relevant product attributes (left table) and their synergies (right table) for the (1) net-zero persona.

Bundle attribute	Capacity value
organic (z_1)	0
lowSalt (h_5)	0
noAdditives (h_3)	0
bundleCardinality (z_6)	0.1
sweetenerFree (h_4)	0.1
lowtransFat (h_2)	0.2
sugarFree (h_1)	0.3

Bundle attribute	Capacity value
(h_1, h_5)	0.4
(h_1, h_4)	0.5
(h_1, h_2)	0.6
(h_1, h_2, h_4)	0.7
(h_1, h_2, h_4, z_1)	0.8
$(h_1, h_2, h_4, h_5, z_1)$	0.9
$(h_1, h_2, h_3, h_4, h_5, z_1)$	1

Figure S4. The capacity values μ adopted to formalize the relevant product attributes (left table) and their synergies (right table) for the (2) healthy persona.

Bundle attribute	Capacity value
itemDissimilarity (s_1)	0
bundleCardinality (z_6)	0.1
globalRelativeFreq (s_4)	0.1
userNovelty (s_2)	0.2
userCategoryNovelty (s_3)	0.3

Bundle attribute	Capacity value
(s_1, s_2)	0.3
(s_1, s_3)	0.4
(s_2, s_4)	0.5
(s_3, s_4)	0.7
(s_2, s_3, s_4)	0.7
(s_1, s_2, s_3, s_4)	1

Figure S5. The capacity values μ adopted to formalize the relevant product attributes (left table) and their synergies (right table) for the (3) serendipitous persona.

Bundle attribute	Capacity value
FreshProducts (l_4)	0
ethicalWorkCertifications (l_5)	0
supplyChainTraceability (l_6)	0.1
vendorFromSameRegion (l_2)	0.1
sameProductionRegion (l_1)	0.1
homeMade products (l_3)	0.2

Bundle attribute	Capacity value
(l_1, l_2)	0.3
(l_3, l_6)	0.4
(l_3, l_5, l_6)	0.6
(l_3, l_4, l_5, l_6)	0.7
(l_1, l_2, l_3)	0.7
$(l_1, l_2, l_3, l_4, l_5, l_6)$	1

Figure S6. The capacity values μ adopted to formalize the relevant product attributes (left table) and their synergies (right table) for the (4) local SME-focused persona.

Bundle attribute	Capacity value
compostablePackaging (z_4)	0
organicProducts (z_1)	0
homemadeProducts (l_3)	0.1
supplyChainTraceability (l_6)	0.2
ethicalWorkCertifications (l_5)	0.2

Bundle attribute	Capacity value
(l_5, l_6)	0.6
(l_3, l_5, l_6)	0.8
(l_3, l_5, l_6, z_1)	0.9
$(l_3, l_5, l_6, z_1, z_4)$	1

Figure S7. The capacity values μ adopted to formalize the relevant product attributes (left table) and their synergies (right table) for the (5) ethical persona.
